# Supplementary material for: Signalome-wide assessment of host cell response to hepatitis C virus
Source: Nat Commun. 2017 May 8;8:15158. doi: 10.1038/ncomms15158 (PMC5424167; doi:10.1038/ncomms15158)
Supplement: Supplementary Information — Supplementary Figures, Supplementary Note and Supplementary References [file ncomms15158-s1.pdf]

Supplementary Figures

Supplementary Figure 1. Antibody microarray analysis of Huh7.5.1 cells transfected with HCV genome.

A) Huh7.5.1 cells were transfected with ΔE1E2 transcripts (right panel) as described in Methods. 24h post-transfection, HCV NS5A was detected by the 9E10 antibody and visualized by anti-mouse conjugated with Alexa 488 (green). Nuclei were stained with DAPI (blue). Left panel: Negative control. Cells treated with transfection reagent alone, in the absence of ΔE1E2 transcripts.

B) Heat map depicting the modulation of the 103 proteins that were identified in the Kinexus microarray experiment as differentially expressed (or differentially phosphorylated) from the control (untransfected) cells in at least one time point. The up-regulated protein expression or phosphorylation levels (Z-Score Ratios) in the transfected (versus control) samples are represented in red, while down-regulated levels are in green. The columns at the left side indicate the time point of differential regulation; colours are explained in the graphical legends at the top. Cell factors have been clustered based on their Z-rations, and each cell factor information follows Uniprot accession number, symbol(s), and phosphorylation sites. The scale bar (bottom right) represents 500µm.

A

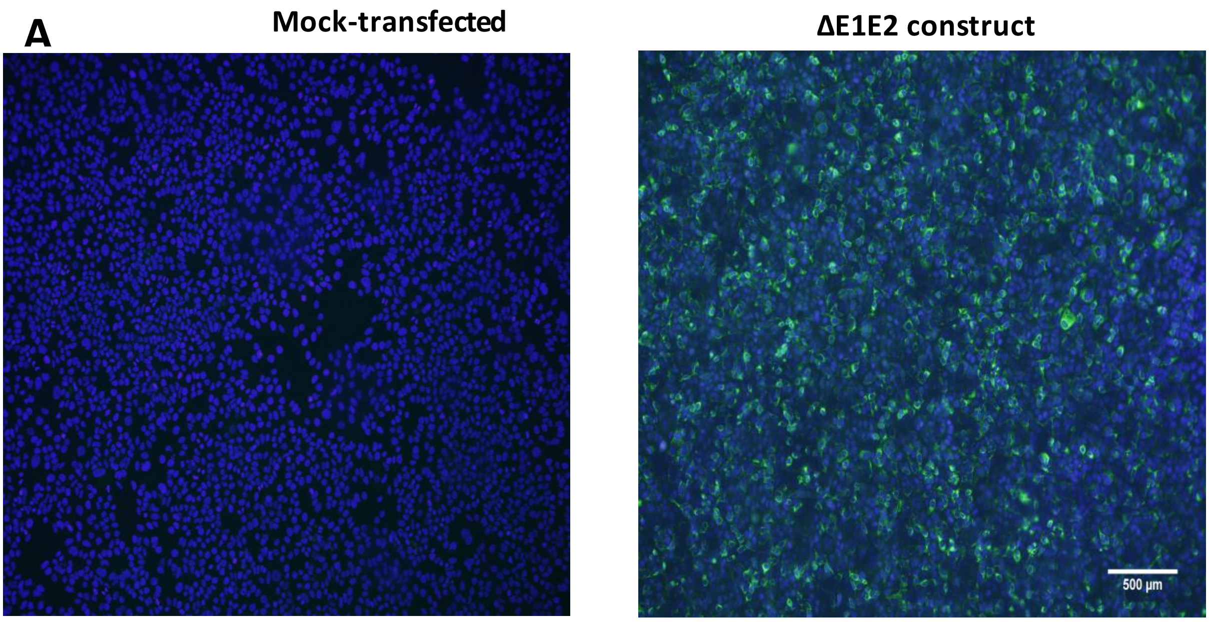

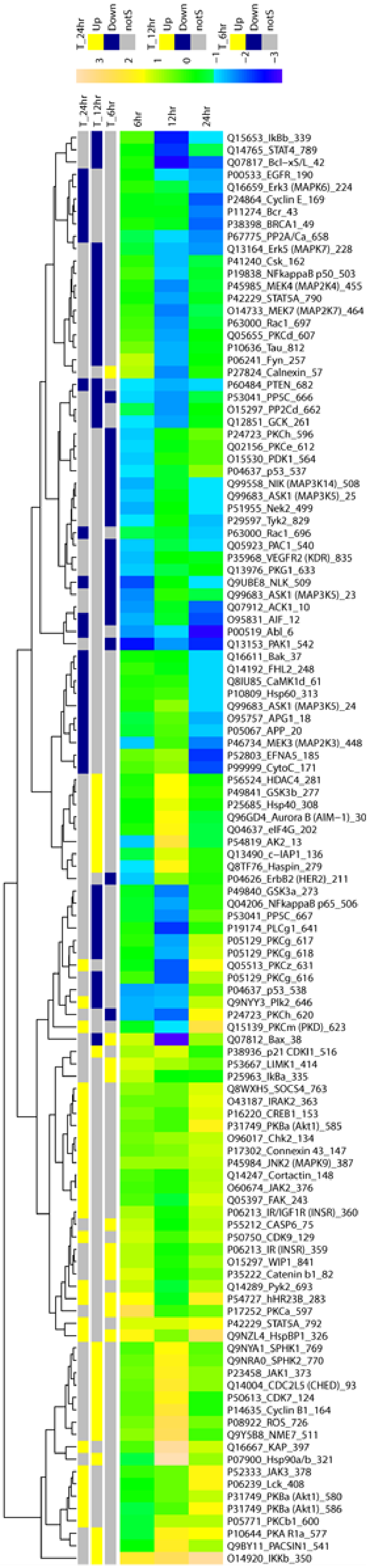

Supplementary Figure 2. Heat map depicting the modulation of the 103 proteins identified with de-regulation of expression and/or phosphorylation in at least one time point. The up-regulated protein expression or phosphorylation levels (Z-Score Ratios) in the treated samples compared to the control sample is represented in red, while down-regulated levels is in green. Cell factors have been clustered based on their Z-rations, and each cell factor information follows Uniprot accession number, symbol(s), and phosphorylation sites. For some factors, more than one antibody has been used.

Supplementary Figure 3. Flowchart of the siRNA gene silencing experiments. SMARTpool siRNA targeting four distinct sites of each transcript by four distinct siRNAs in each pool were transfected into the cells. The cells were then infected with reporter HCV and the effect of gene silencing on virus replication was determined. Cell viability was measured as described in Methods.

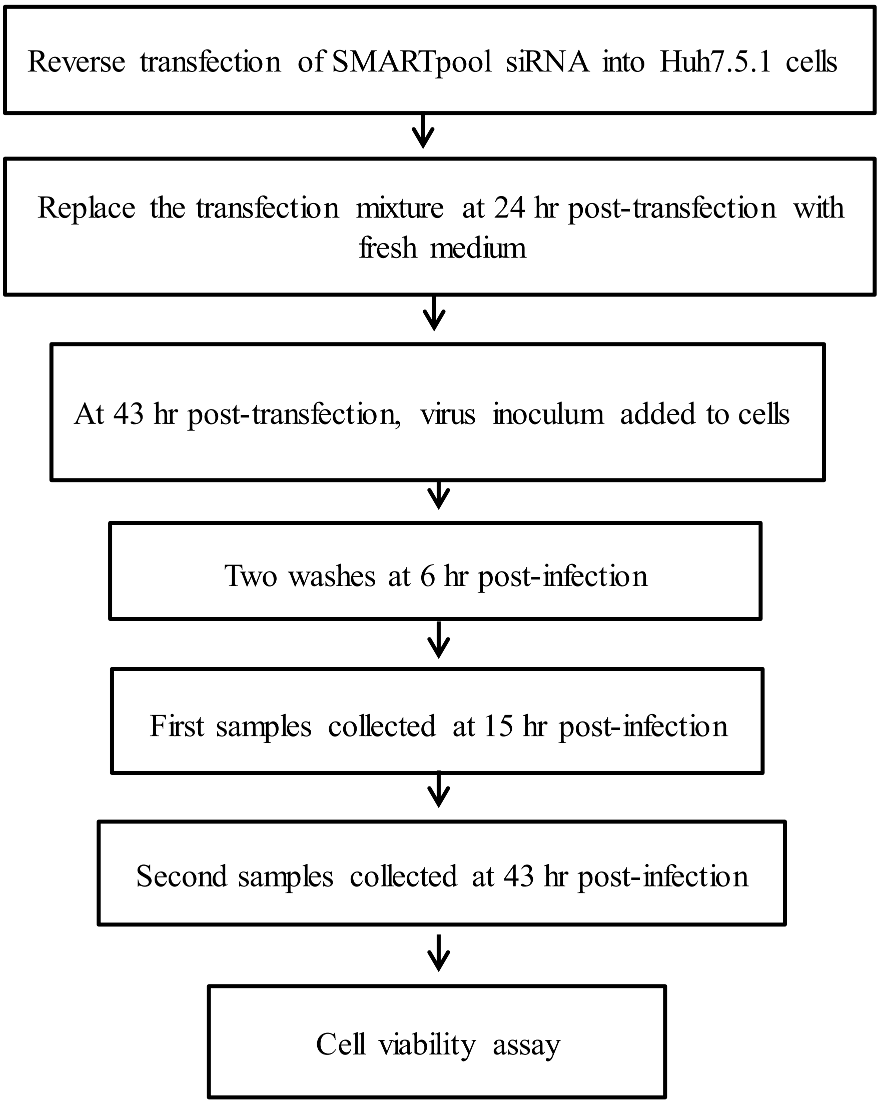

Supplementary Figure 4. siRNA-mediated MAP4K2 silencing.

A) Huh7.5.1 cells were transfected with SMARTpool and individual MAP4K2 individual siRNA (Supplementary Data 5) prior to Western blot analysis (see Methods). Specific MAP4K2 antibody was used to detect the MAP4K2 protein. Actin was used as a loading control.

B) The intensity of each protein signal was measured by Image Lab™ software; values for MAP4K2 were normalized to those of actin and then to the control scrambled OTP-NT value set at 100%. Novex® Sharp Pre-stained Protein Standard (Novex) was used as protein size marker.

A

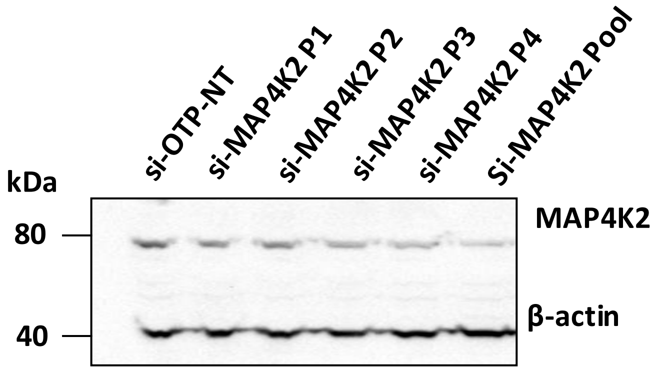

B

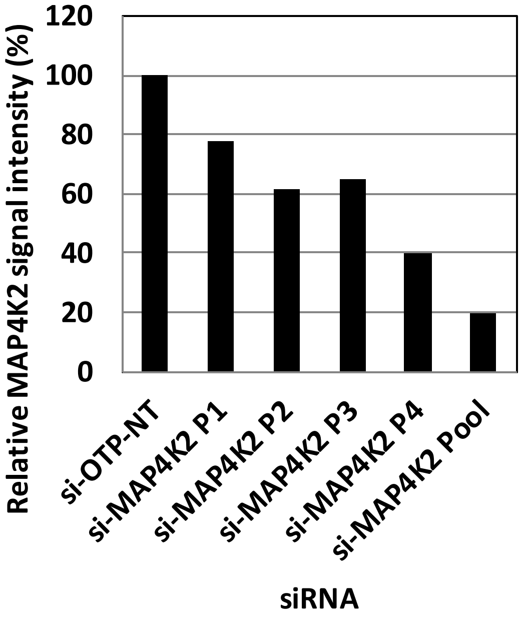

Supplementary Figure 5. Effects of TL4-12 on HCV replication.

A) Huh7.5.1 cells were treated with 6 µM and 12 µM TL4-12 and subsequently infected with HCV prior to Western blot analysis. Untreated, mock-infected, and untreated HCV-infected cells (DMSO-treated) acted as controls. The 9E10 antibody was used to detect the HCV NS5A protein as a measure of viral gene product expression. Actin was used as a loading control. Novex® Sharp Pre-stained Protein Standard (Novex) was used as protein size marker. B) The intensity of each protein signal was measured by Image Lab™ software; values for NS5A were normalized to those of actin and then to the mock-treated value set at 100%. Bars represent standard deviation of two independent experiments. C) Huh7.5.1 cells were treated the inhibitor and infected with HCV for 24h prior to immunofluorescence assay. Untreated, HCV-infected (DMSO-treated) and untreated, non-infected (Mock-infected) cells acted as negative controls. HCV NS5A was detected by the 9E10 antibody and visualized by anti-mouse Alexa 488 (green). Nuclei were stained with DAPI (blue). The scale bar (bottom right) represents 100µm.

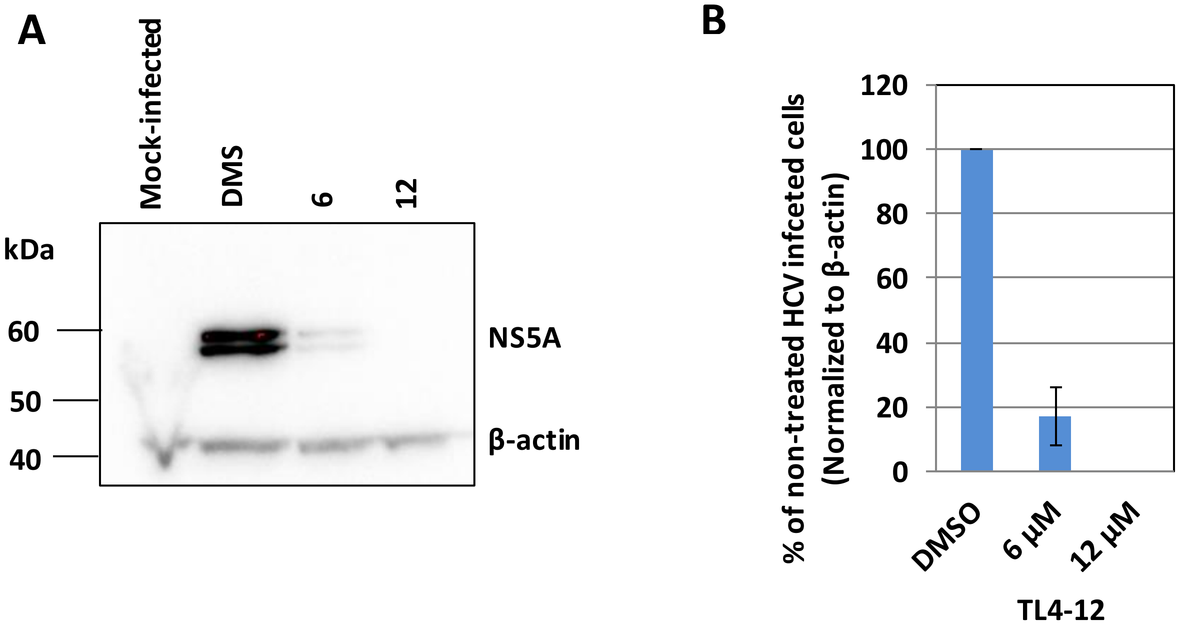

C

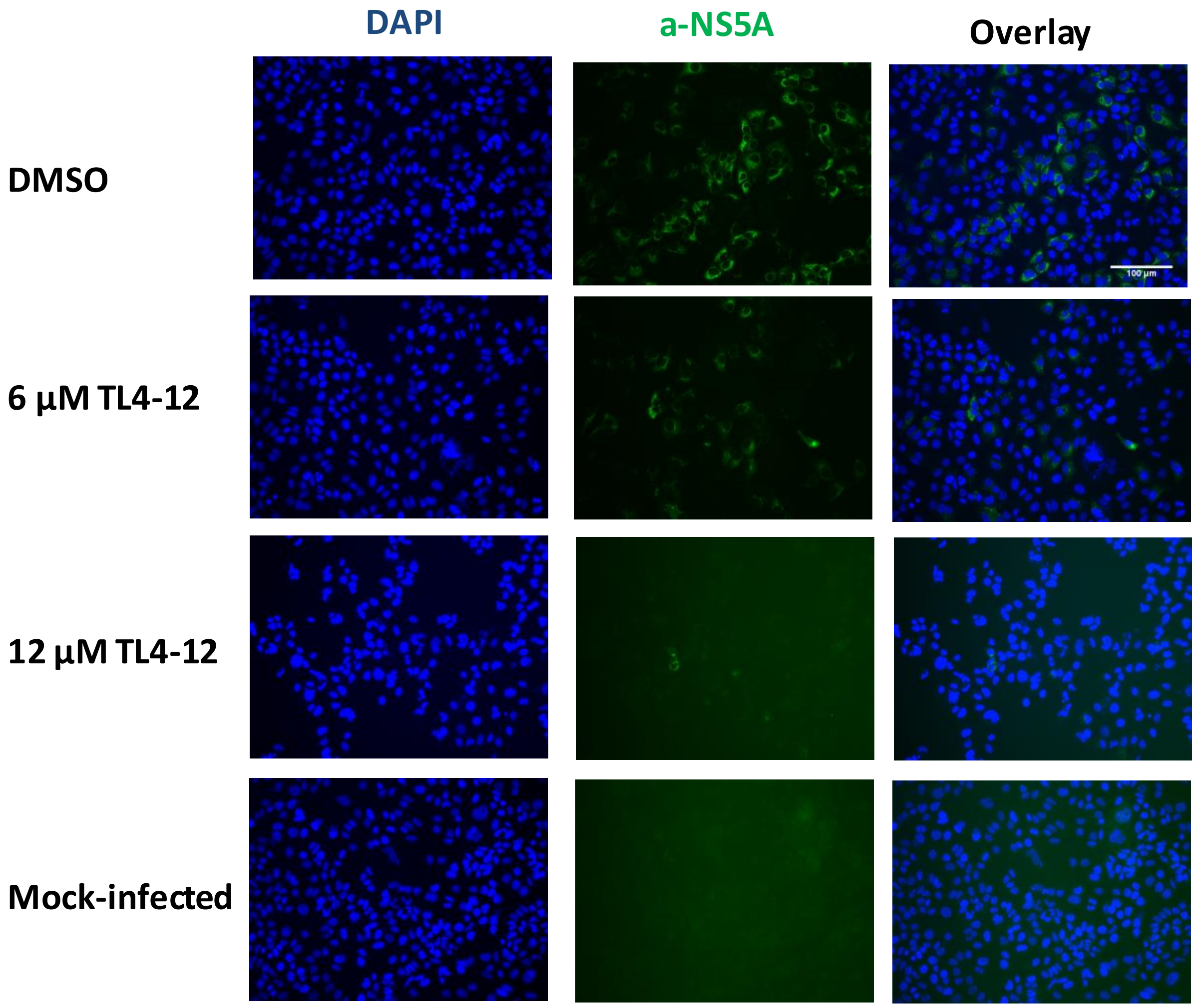

Supplementary Figure 6. Toxicity of TL4-12 for Huh7.5.1 cells.

Different concentrations of the compound were added to overnight cultured 5 x 10^3 cells per each well of a 96-well plate (triplicates). Corresponding concentrations of vehicle DMSO were used as negative control. After an additional 48h incubation in the presence of the compound, the viability of cells was tested by PrestoBlue® Cell Viability reagent (Thermofisher) as described in Methods.

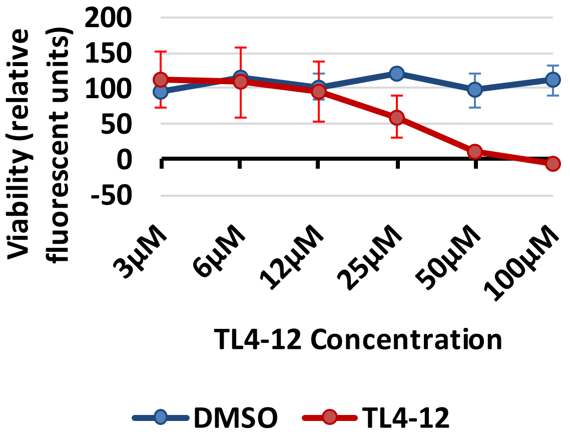

Supplementary Note 1

Additional pathways

Insulin/PI3K/AKT signalling pathway. Ligand binding to the insulin receptor leads to activation of the AKT protein kinase and of PI3K (phosphatidylinositol 3-kinase), which then mediate metabolic and cell survival pathways [10]. The insulin receptor is phosphorylated at tyrosine 999 at 6h post transfection and at tyrosine 1189/1190 at 24h post transfection (Supplementary Data 1 and Supplementary Data 3; Supplementary Fig. 7). Silencing of the insulin receptor only slightly reduced virus replication (Supplementary Data 6, Supplementary Fig. 7). Of the three AKT isoforms (AKT1-3), only AKT1 was significantly upregulated at 24h (Supplementary Data 3). Phosphorylation of AKT1 at threonine 308 (AKT T308p) decreased 24h post-transfection, while a significant increase in phosphorylation of serine 473 and tyrosine 474 (AKT1 S473p and Y474p) was observed. Phosphorylation of AKT T308 promotes glucose uptake. AKT Y474 phosphorylation is partially required for AKT activation [11]. The results are consistent with the fact that HCV patients are at high risk of developing type II diabetes [12]. Additionally, PDK1 (3-phosphoinositide-dependent protein kinase-1), which activates AKT1 through phosphorylation of T308, did not show a decrease in abundance or in phosphorylated state at any time point. Consistent with the absence of mobilisation of the kinase following infection, silencing PDK1 did not affect virus replication (Supplementary Data 6, Supplementary Fig. 7). Interestingly, the other kinase known to phosphorylate AKT1 at S473, mTORC2 (mammalian target of rapamycin complex 2), also remained unaltered at all time points. However, the levels of total and phosphorylated form of the Phosphatase and Tensin Homolog (PTEN) show a significant reduction at 12h and 24h; in view of the absence of modulation in the levels of the activating kinases, it is possible that a decrease of PTEN-mediated phosphatase activity is responsible for the increase of AKT1 S473 [13]. Previous reports indicated that the HCV core protein induces lipogenesis through a reduction in total PTEN level [14,15], and it has been demonstrated that infection with HCV genotypes 1 and 2, as well as expression of the HCV core protein, induce phosphorylation of AKT at S473 but not at T308 [16]. A recent study confirmed the reduction of PTEN by HCV and showed that phosphorylation of insulin receptor substrate-1 (IRS-1) is regulated by PTEN in HCV-infected cells [17]. We now show that phosphorylation of IRS-1 moderately increased on Y612 and Y1179 but decreased on S312 and S639. Our results indicate that silencing AKT1 enhances HCV entry/viral genome replication, whereas silencing PTEN decreases virus replication (Table S7), suggesting opposing roles for AKT1 and PTEN in the process. However, our results do not exclude a direct role for PTEN in HCV replication. Interestingly, the suppressive role of PI3K/AKT in virus replication has been also demonstrated for hepatitis B virus (HBV) [18]. AKT is a major regulator of cell survival: it phosphorylates the apoptotic modulator BAD (BCL2-antagonist cell death) at serine 136 (its pro-survival configuration) and also phosphorylates CREB1 (cAMP response element binding protein), which has a positive regulatory role in cell survival [19]. Consistent with our observation regarding AKT modulation, the level of phosphorylated CREB1 (S129, S133) shows a

significant increase 24h post-transfection and its silencing reduced virus replication (Tables S3 and S6, Supplementary Fig. 7). This is the first report suggesting a role for cAMP/CREB1 in HCV replication.

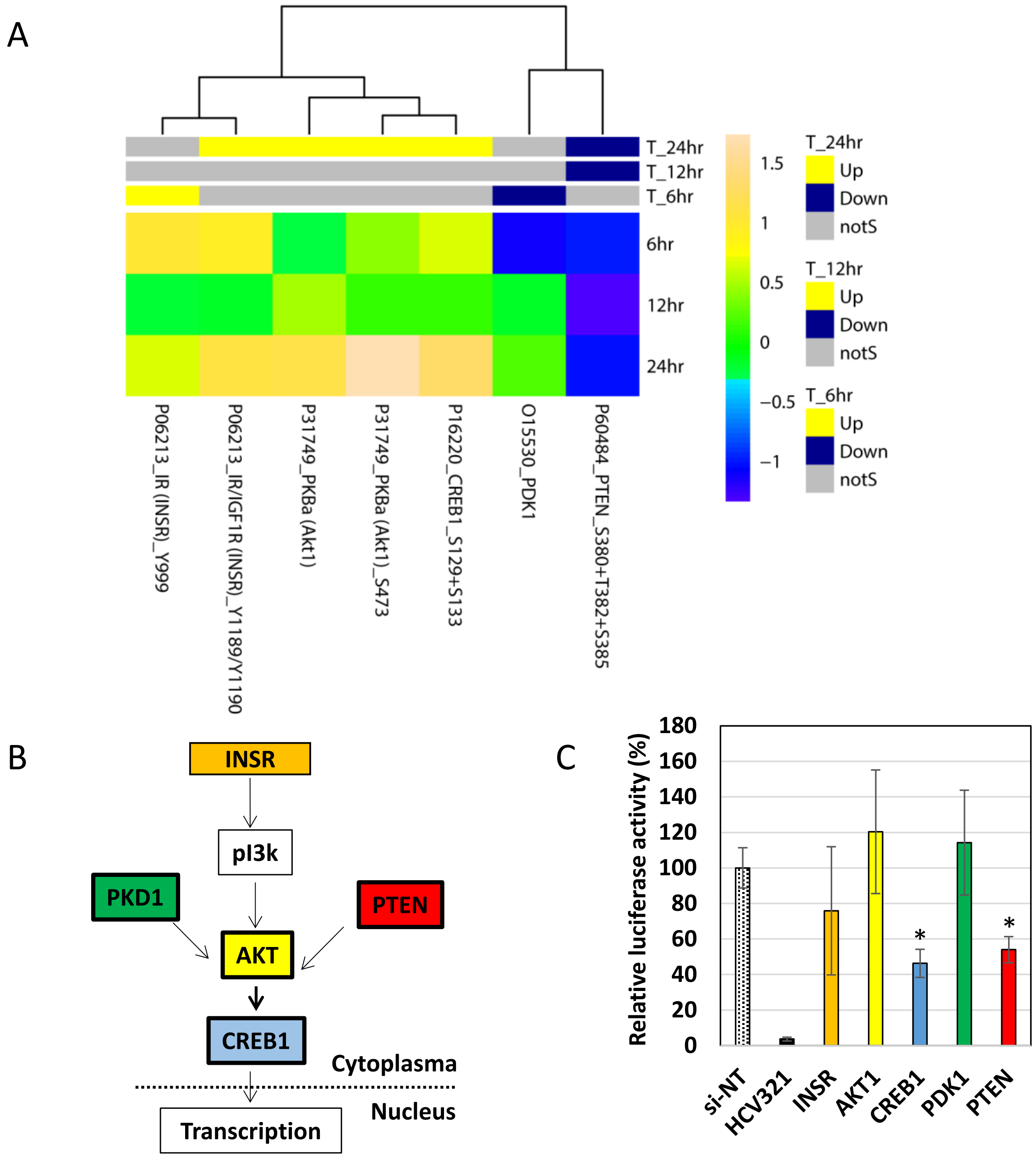

Supplementary Figure 7. Impact of HCV on the insulin/PI3K/AKT pathway components on HCV replication. A) Up and down-regulation of cell factors, determined by the antibody microarray experiment. Factors with a Z ratio higher than 1.25 are considered significant (see Methods) (B) A schematic figure of simplified insulin/PI3K/AKT pathway. Colour boxes represent factors shortlisted for siRNA validation following the microarray experiment. C) Effect of silencing of the insulin/PI3K/AKT pathway components on HCV replication. Following silencing the target genes for 43h, cells were infected with a reporter virus containing a Renilla luciferase gene. The luciferase activity of each well was measured and normalized to its viability and negative control si-OTP-NT set at 100%. The error bars represent standard deviation of at least 4 wells. P values were calculated as described in Methods; significant variations (P<0.05) are depicted by

Wnt/β-catenin pathway. This pathway is involved in early embryogenesis and is activated in several cancers [35]. β-catenin, a key mediator of this pathway, translocates into the nucleus upon stimulation and triggers transcription of its target cell genes. The level of phosphorylated β-catenin (S552) increases after infection with HCV, and this mediates activation of AKT [36]. Another important component of the Wnt/β-catenin pathway is the nemo-like kinase (NLK) that, through phosphorylation of the TCF transcription factor (a β-catenin partner), promotes or inhibits the transcription of target genes [37]. Consistent with published data that the HCV core protein activates the canonical Wnt signalling pathway [38], we observed an increase in the expression of β-catenin 6h post transfection (Supplementary Data 1). Active β-catenin becomes inactivated as a consequence of its phosphorylation by glycogen synthase kinase-3β (GSK-3β), and is thereafter quickly degraded. GSK-3β levels were high at 12h but low at 24h. NLK abundance decreased at time point 6h and remained lower than the control throughout the study (Supplementary Data 1-3). Silencing NLK and β-catenin supressed virus replication (Supplementary Data 6, Supplementary Fig. 8), further suggesting that Wnt signalling is important for virus replication.

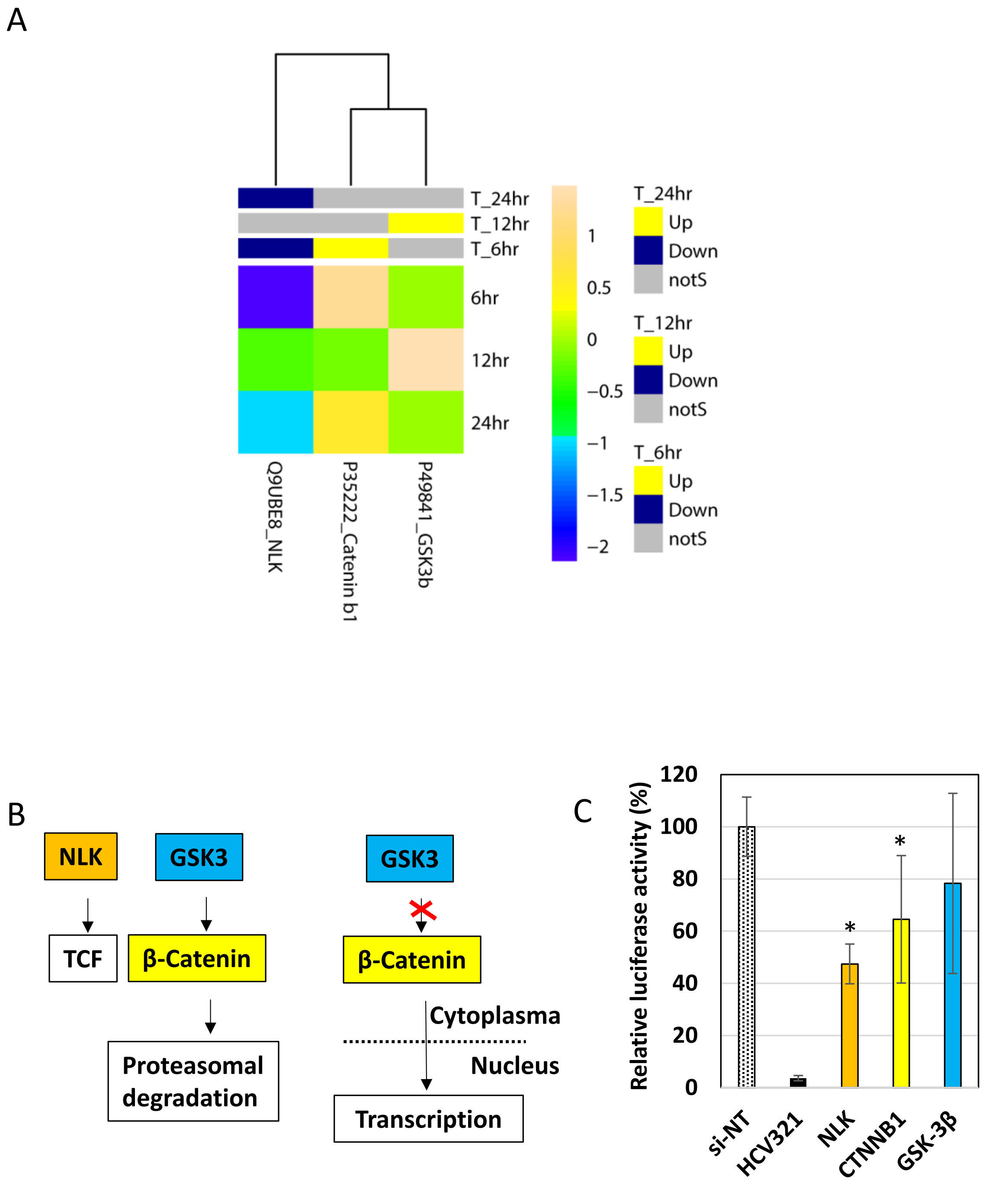

Supplementary Figure 8. Wnt/ β-catenin pathway. A) Up and down-regulation of cell factors determined by antibody microarray experiment. Factors with a Z ratio higher than 1.25 are considered significant (see Methods) (B) A schematic figure of simplified Wnt/β-catenin pathway. Colour boxes represent factors shortlisted for siRNA validation following the microarray experiment. C) Effect of silencing of the Wnt/β-catenin pathway components on HCV replication. Following silencing the target genes for 43h, cells were infected with a reporter virus containing a Renilla luciferase gene. The luciferase activity of each well was measured and normalized to its viability and negative control si-OTP-NT set at 100%. The error bars represent standard deviation of at least 4 wells. P values were calculated as described in Methods; significant variations ($P<0.05$) are depicted by an

Apoptosis. As alluded to above, apoptosis is one of the major pathways that were modulated across the 3 time points (Supplementary Data 1-3). Apoptosis is a very complex programmed, host cell innate response to pathogens. As shown in Table S4, apoptosis is the first pathway whose several cell mediators have been modulated following transfection of viral RNA. Several cell factors transmit apoptosis-inducing signals to the nucleus. Bcl2-associated X protein (Bax) and Bcl2-antagonist/killer 1 (Bak1) are two pro-apoptotic factors that are inserted into mitochondrial membrane upon activation, causing cytochrome C release that, in turn, activates caspases, which are major effectors of apoptosis. Our results indicate that Bax was upregulated 6h post transfection (Supplementary Data 1), in line with published data indicating that Bax is induced by HCV infection [39]. 12h post-transfection, Bax became the most drastically down-regulated protein, suggesting suppression of apoptosis at this time point. In line with this observation, Bak was also significantly down-regulated at 24h post-transfection (Supplementary Data 3). Surprisingly, silencing of Bax and Bak slightly reduced and stimulated virus replication, respectively (Supplementary Data 6, Fig. 7). Interestingly, similar to Bak, Bcl-xl (see above) was also supressed at 12h post transfection, suggesting that a fine-tuned balance between expression of Bak and Bcl-xl is required for optimal HCV replication. The reduction in Bcl-xl levels is unexpected because the phosphorylated active form of the STAT5A transcription factor, an activator of Bcl-xl gene transcription, is significantly increased at 24h (Supplementary Data 3). It is possible that the expression of Bcl-xl is supressed post-transcriptionally. Collectively, our data suggest that at 24h post-transfection, downregulation of Bak and Bcl-xl is important for optimal replication of HCV.
c-IAP inhibits apoptosis by destabilizing the second mitochondria-derived activator of caspases (smac) [40]. It increased at 12h but its silencing did not affect virus replication (Supplementary Fig. 9). Overall, it is clear that HCV infection leads to interference with apoptosis and promotes cell survival.

Cell cycle progression. Our microarray data pointed to the involvement of cell division control elements, such as the Polo-like kinase 2 (PLK2), which regulates G1/S transition, and the mitotic cylin B1 (Supplementary Data 1, Supplementary Fig. 10). Silencing either PLK2 or CDK13 (also known as CDC2L5 and CHED) reduced virus replication (Supplementary Data 6, Supplementary Fig. 10). On the other hand silencing cell division regulators such as ERBB2-regulated CDKN1A (a cyclin-dependent kinase inhibitor, also known as p21 CDKI1) [41] up-regulated virus replication. ERBB2 was downregulated at 6h. CDKN1A was up-regulated at 12h.

A

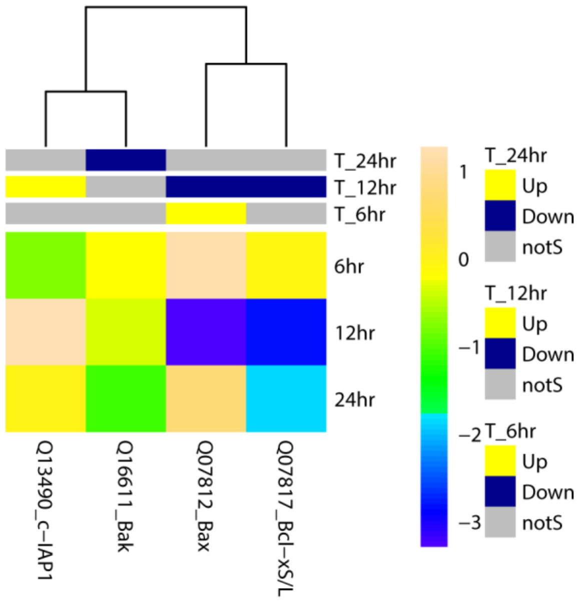

B

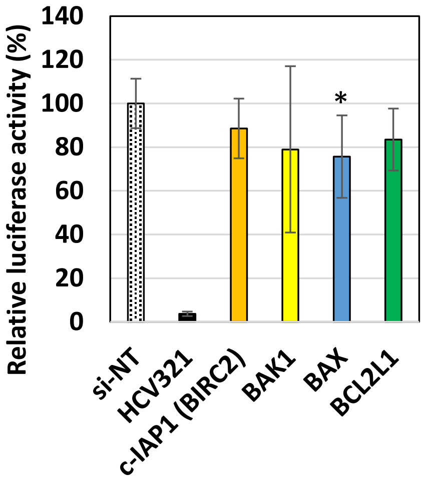

Supplementary Figure 9. Modulation of apoptosis by HCV at different time points post-transfection. A) Up and down-regulation of cell factors determined by antibody microarray experiment. Factors with a Z ratio higher than 1.25 are considered significant (see Methods) (B) Effect of silencing of the identified genes involved in cell apoptosis on HCV replication. Following silencing the target genes for 43h, cells were infected with a reporter virus containing a Renilla luciferase gene. The luciferase activity of each well was measured and normalized to its viability and negative control si-OTP-NT set at 100%. The error bars represent standard deviation of at least 4 wells. P values were calculated as described in Methods; significant variations ($P<0.05$) are depicted by an asterisk.

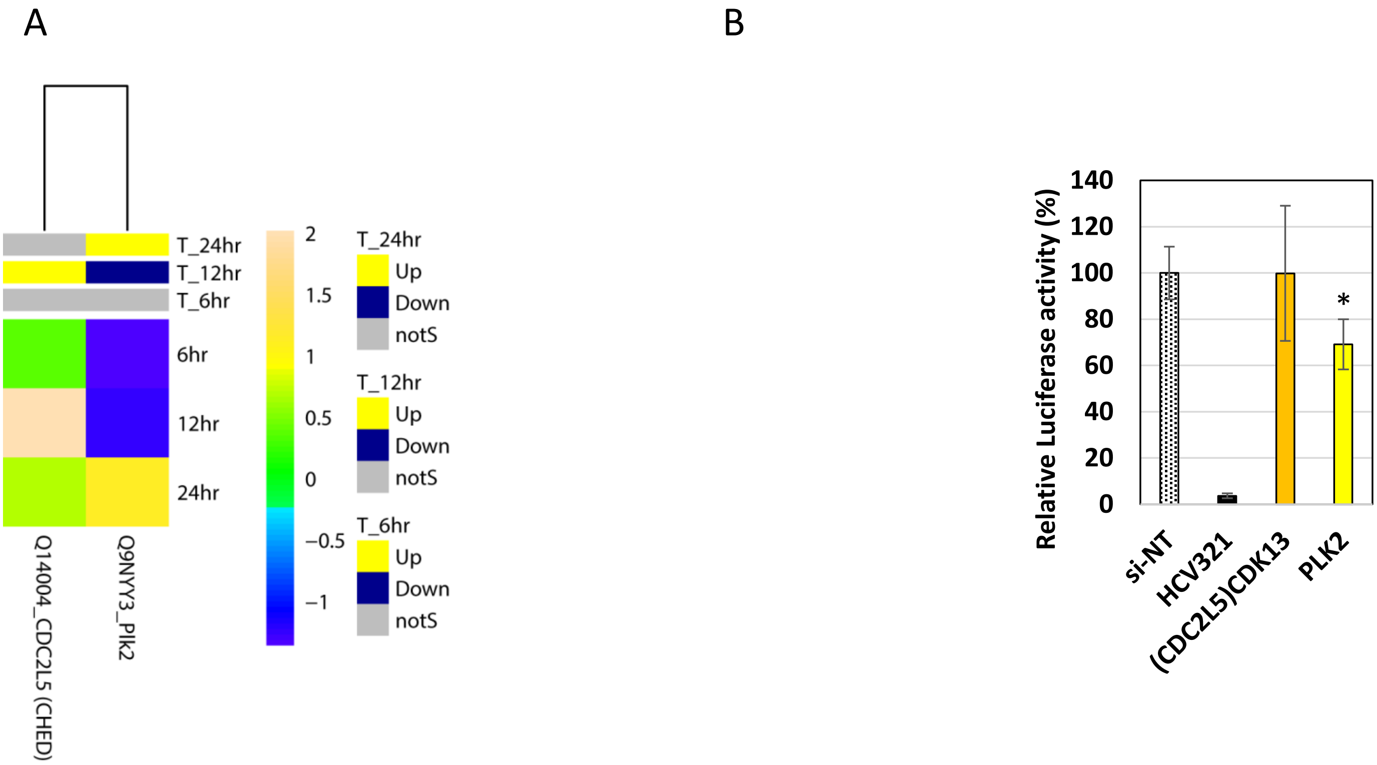

Supplementary Figure 10. Modulation and importance of cell factors in cell cycle progression in HCV replication. A) Up and down-regulation of cell factors determined by antibody microarray experiment. Factors with a Z ratio higher than 1.25 are considered significant (see Methods) (B) Effect of gene silencing on HCV replication. Following silencing the target genes for 43h, cells were infected with a reporter virus containing a Renilla luciferase gene. The luciferase activity of each well was measured and normalized to its viability and negative control si-OTP-NT set at 100%. The error bars represent standard deviation of at least 4 wells. P values were calculated as described in Methods; significant variations (P<0.05) are depicted by an asterisk.

Supplementary References

1. Hinz, M. & Scheidereit, C. The IkappaB kinase complex in NF-kappaB regulation and beyond. *EMBO reports* **15**, 46-61 (2014).
2. Hayden, M.S. & Ghosh, S. NF-kappaB, the first quarter-century: remarkable progress and outstanding questions. *Genes & development* **26**, 203-234 (2012).
3. Park, J., *et al.* Hepatitis C virus infection enhances TNFalpha-induced cell death via suppression of NF-kappaB. *Hepatology* **56**, 831-840 (2012).
4. Li, Q., Pene, V., Krishnamurthy, S., Cha, H. & Liang, T.J. Hepatitis C virus infection activates an innate pathway involving IKK-alpha in lipogenesis and viral assembly. *Nature medicine* **19**, 722-729 (2013).
5. Zhang, L., *et al.* The modulation of hepatitis C virus 1a replication by PKR is dependent on NF-kB mediated interferon beta response in Huh7.5.1 cells. *Virology* **438**, 28-36 (2013).
6. Sun, S.C. The noncanonical NF-kappaB pathway. *Immunological reviews* **246**, 125-140 (2012).
7. Varfolomeev, E., *et al.* IAP antagonists induce autoubiquitination of c-IAPs, NF-kappaB activation, and TNFalpha-dependent apoptosis. *Cell* **131**, 669-681 (2007).
8. Lee, S., Challa-Malladi, M., Bratton, S.B. & Wright, C.W. Nuclear factor-kappaB-inducing kinase (NIK) contains an amino-terminal inhibitor of apoptosis (IAP)-binding motif (IBM) that potentiates NIK degradation by cellular IAP1 (c-IAP1). *The Journal of biological chemistry* **289**, 30680-30689 (2014).
9. Zhu, H. & Liu, C. Interleukin-1 inhibits hepatitis C virus subgenomic RNA replication by activation of extracellular regulated kinase pathway. *Journal of virology* **77**, 5493-5498 (2003).
10. Niswender, K.D., *et al.* Insulin activation of phosphatidylinositol 3-kinase in the hypothalamic arcuate nucleus: a key mediator of insulin-induced anorexia. *Diabetes* **52**, 227-231 (2003).
11. Conus, N.M., Hannan, K.M., Cristiano, B.E., Hemmings, B.A. & Pearson, R.B. Direct identification of tyrosine 474 as a regulatory phosphorylation site for the Akt protein kinase. *J Biol Chem* **277**, 38021-38028 (2002).
12. Mehta, S.H., *et al.* Prevalence of type 2 diabetes mellitus among persons with hepatitis C virus infection in the United States. *Annals of internal medicine* **133**, 592-599 (2000).
13. Zhao, X., *et al.* Phosphoinositide-dependent kinase 1 and mTORC2 synergistically maintain postnatal heart growth and heart function in mice. *Molecular and cellular biology* **34**, 1966-1975 (2014).
14. Clement, S., *et al.* Down-regulation of phosphatase and tensin homolog by hepatitis C virus core 3a in hepatocytes triggers the formation of large lipid droplets. *Hepatology* **54**, 38-49 (2011).
15. Peyrou, M., *et al.* PTEN protein phosphatase activity regulates hepatitis C virus secretion through modulation of cholesterol metabolism. *Journal of hepatology* **59**, 420-426 (2013).
16. Banerjee, S., *et al.* Hepatitis C virus core protein upregulates serine phosphorylation of insulin receptor substrate-1 and impairs the downstream akt/protein kinase B signaling pathway for insulin resistance. *Journal of virology* **82**, 2606-2612 (2008).
17. Gao, T.T., Qin, Z.L., Ren, H., Zhao, P. & Qi, Z.T. Inhibition of IRS-1 by hepatitis C virus infection leads to insulin resistance in a PTEN-dependent manner. *Virology journal* **12**, 12 (2015).
18. Guo, H., *et al.* Regulation of hepatitis B virus replication by the phosphatidylinositol 3-kinase-akt signal transduction pathway. *Journal of virology* **81**, 10072-10080 (2007).

19. Bonni, A., *et al.* Cell survival promoted by the Ras-MAPK signaling pathway by transcription-dependent and -independent mechanisms. *Science* **286**, 1358-1362 (1999).
20. Darnell, J.E., Jr. STATs and gene regulation. *Science* **277**, 1630-1635 (1997).
21. Dumon, S., *et al.* IL-3 dependent regulation of Bcl-xL gene expression by STAT5 in a bone marrow derived cell line. *Oncogene* **18**, 4191-4199 (1999).
22. Matikainen, S., *et al.* Interferon-alpha activates multiple STAT proteins and upregulates proliferation-associated IL-2Ralpha, c-myc, and pim-1 genes in human T cells. *Blood* **93**, 1980-1991 (1999).
23. Bautista, D., Bermudez-Silva, F.J., Lasarte, J.J., Rodriguez-Fonseca, F. & Baixeras, E. Liver expression of proteins controlling interferon-mediated signalling as predictive factors in the response to therapy in patients with hepatitis C virus infection. *The Journal of pathology* **213**, 347-355 (2007).
24. Hong, S. & Laimins, L.A. The JAK-STAT transcriptional regulator, STAT-5, activates the ATM DNA damage pathway to induce HPV 31 genome amplification upon epithelial differentiation. *PLoS pathogens* **9**, e1003295 (2013).
25. Watanabe, S., Zeng, R., Aoki, Y., Itoh, T. & Arai, K. Initiation of polyoma virus origin-dependent DNA replication through STAT5 activation by human granulocyte-macrophage colony-stimulating factor. *Blood* **97**, 1266-1273 (2001).
26. Cheshenko, N., *et al.* Herpes simplex virus triggers activation of calcium-signaling pathways. *The Journal of cell biology* **163**, 283-293 (2003).
27. Keay, S., Baldwin, B.R., Smith, M.W., Wasserman, S.S. & Goldman, W.F. Increases in [Ca2+]i mediated by the 92.5-kDa putative cell membrane receptor for HCMV gp86. *The American journal of physiology* **269**, C11-21 (1995).
28. Zhou, Y., Frey, T.K. & Yang, J.J. Viral calciomics: interplays between Ca2+ and virus. *Cell calcium* **46**, 1-17 (2009).
29. Scherbik, S.V. & Brinton, M.A. Virus-induced Ca2+ influx extends survival of west nile virus-infected cells. *Journal of virology* **84**, 8721-8731 (2010).
30. Kadamur, G. & Ross, E.M. Mammalian phospholipase C. *Annual review of physiology* **75**, 127-154 (2013).
31. Dong, Y., Zeng, C.Q., Ball, J.M., Estes, M.K. & Morris, A.P. The rotavirus enterotoxin NSP4 mobilizes intracellular calcium in human intestinal cells by stimulating phospholipase C-mediated inositol 1,4,5-trisphosphate production. *Proceedings of the National Academy of Sciences of the United States of America* **94**, 3960-3965 (1997).
32. Bhowmick, R., Banik, G., Chanda, S., Chattopadhyay, S. & Chawla-Sarkar, M. Rotavirus infection induces G1 to S phase transition in MA104 cells via Ca(+)(2)/Calmodulin pathway. *Virology* **454-455**, 270-279 (2014).
33. Sakagami, H., *et al.* Prominent expression and activity-dependent nuclear translocation of Ca2+/calmodulin-dependent protein kinase Idelta in hippocampal neurons. *The European journal of neuroscience* **22**, 2697-2707 (2005).
34. Zen, K., Biwersi, J., Periasamy, N. & Verkman, A.S. Second messengers regulate endosomal acidification in Swiss 3T3 fibroblasts. *The Journal of cell biology* **119**, 99-110 (1992).
35. Hoppler, S. & Kavanagh, C.L. Wnt signalling: variety at the core. *Journal of cell science* **120**, 385-393 (2007).

36. Bose, S.K., Meyer, K., Di Bisceglie, A.M., Ray, R.B. & Ray, R. Hepatitis C virus induces epithelial-mesenchymal transition in primary human hepatocytes. *Journal of virology* **86**, 13621-13628 (2012).
37. Ishitani, T. & Ishitani, S. Nemo-like kinase, a multifaceted cell signaling regulator. *Cellular signalling* **25**, 190-197 (2013).
38. Liu, J.*, et al.* Hepatitis C virus core protein activates Wnt/beta-catenin signaling through multiple regulation of upstream molecules in the SMMC-7721 cell line. *Archives of virology* **156**, 1013-1023 (2011).
39. Deng, L.*, et al.* Hepatitis C virus infection induces apoptosis through a Bax-triggered, mitochondrion-mediated, caspase 3-dependent pathway. *Journal of virology* **82**, 10375-10385 (2008).
40. Hu, S. & Yang, X. Cellular inhibitor of apoptosis 1 and 2 are ubiquitin ligases for the apoptosis inducer Smac/DIABLO. *The Journal of biological chemistry* **278**, 10055-10060 (2003).
41. Yu, D.*, et al.* Overexpression of ErbB2 blocks Taxol-induced apoptosis by upregulation of p21Cip1, which inhibits p34Cdc2 kinase. *Molecular cell* **2**, 581-591 (1998).
