# Supplementary material for: Signalome-wide assessment of host cell response to hepatitis C virus
Source: Nat Commun. 2017 May 8;8:15158. doi: 10.1038/ncomms15158 (PMC5424167; doi:10.1038/ncomms15158)
Supplement: Supplementary Data 9 — Oligonucleotides used in qRT-PCR experiments [file ncomms15158-s10.docx]

| **Oligonucleotide** | **Nucleotide sequence** |
| --- | --- |
| HCV forward primer (positions 130-146) | 5'-CGGGAGAGCCATAGTGG-3' |
| HCV reverse primer (positions 290-272) | 5'-AGTACCACAAGGCCTTTCG-3' |
| HCV probe (positions 148–168) | 56-FAM/CTGCGGAAC/ZEN/CGGTGAGTACAC/3IABkFQ |
| GAPDH forward primer | 5'- CTGCACCACCAACTGCTTAG -3' |
| GAPDH reverse primer | 5'- GTCTTCTGGGTGGCAGTGAT -3' |
| β-actin forward primer | 5'- CTGGCACCACACCTTCTACAATG -3' |
| β-actin reverse primer | 5'- GGTCTCAAACATGATCTGGGTC -3' |

**Supplementary Data 9:** List of oligonucleotides that were used in qRT-PCR experiments. HCV oligonucleotide sequences are conserved among all HCV genotypes (same nucleotide position numbers).
